# Non-Normalizable Quasi-Equilibrium Solution of the Fokker–Planck Equation for Nonconfining Fields

**DOI:** 10.3390/e23020131

**Published:** 2021-01-20

**Authors:** Celia Anteneodo, Lucianno Defaveri, Eli Barkai, David A. Kessler

**Affiliations:** 1 Department of Physics, Pontifícia Universidade Católica do Rio de Janeiro (PUC-Rio), Rio de Janeiro 22541-900, Brazil; lucianno-defaveri@puc-rio.br; 2 Institute of Science and Technology for Complex Systems, Rio de Janeiro 22290-180, Brazil; 3 Department of Physics, Institute of Nanotechnology and Advanced Materials, Bar-Ilan University, Ramat-Gan 52900, Israel; Eli.Barkai@biu.ac.il (E.B.); kessler@dave.ph.biu.ac.il (D.A.K.)

**Keywords:** quasi-equilibrium, Boltzmann-Gibbs regularization, non-confining fields

## Abstract

We investigate the overdamped Langevin motion for particles in a potential well that is asymptotically flat. When the potential well is deep as compared to the temperature, physical observables, like the mean square displacement, are essentially time-independent over a long time interval, the stagnation epoch. However, the standard Boltzmann-Gibbs (BG) distribution is non-normalizable, given that the usual partition function is divergent. For this regime, we have previously shown that a regularization of BG statistics allows for the prediction of the values of dynamical and thermodynamical observables in the non-normalizable quasi-equilibrium state. In this work, based on the eigenfunction expansion of the time-dependent solution of the associated Fokker–Planck equation with free boundary conditions, we obtain an approximate time-independent solution of the BG form, being valid for times that are long, but still short as compared to the exponentially large escape time. The escaped particles follow a general free-particle statistics, where the solution is an error function, which is shifted due to the initial struggle to overcome the potential well. With the eigenfunction solution of the Fokker–Planck equation in hand, we show the validity of the regularized BG statistics and how it perfectly describes the time-independent regime though the quasi-stationary state is non-normalizable.

## 1. Introduction

Systems with interactions that vanish at long distances are ubiquitous in nature, ranging from charged particles to cosmological objects. Here, we investigate this case for a single particle coupled to a thermal heat bath and subject to a potential V(x), which is confining at short distances, but nonconfining otherwise. If the well is deep, such a field can present long-lived quasi-equilibrium states for sufficiently low temperature *T* [1]. Over certain timescales, thermodynamic quantities, e.g., the free energy F, energy *E*, and the entropy S, as well as dynamical ones, e.g., the mean square displacement (MSD), attain a time-independent value and the virial theorem approximately holds, which immediately raises the question about the possibility of using Boltzmann-Gibbs statistics. This is nontrivial, because the expression for the equilibrium probability, for instance, in one dimension, defined as $P_{eq}\left(x\right)=\frac{1}{Z}\;e^{-V\left(x\right)/\left(k_{B}T\right)}$, where $k_{B}$ is the Boltzmann constant, fails due to the diverging of the normalizing partition function in the denominator, $Z=\int_{-\infty }^{\infty }e^{-V\left(x\right)/\left(k_{B}T\right)}\;dx$, for non-confining fields. Here, we will focus our attention on one dimensional systems, where *x* represents the coordinate of the particle; however, the stagnation of the dynamics in a well that is asymptotically flat, for a particle that is described by the Langevin dyanmics, is a general phenomenon that is also found in higher dimensions. The coordinate *x* can represent the distance of a particle from the surface of a membrane, or the coordinate of a particle in an optical trap. Further examples are the hydrogen atom (Coulomb potential) coupled to a thermal bath at temperature *T* [2,3], and the atmosphere in the gravitational field of the earth. What is remarkable is that certain aspects of standard statistical physics and thermodynamics can still be applied through a suitable regularization of $P_{eq}\left(x\right)$ and observable averages, as we have shown in previous work [1], where we developed a general formalism for the problem. This was based on scaling solutions of the Fokker–Planck equation (FPE) for the probability density function (PDF) P(x,t), and alternatively on finite-box solutions. In this paper, we address the problem through the time-dependent solution expressed as an eigenfunction expansion [4,5], and then identifying the non-normalizable quasi-equilibrium (NQE) regime, where these solutions become effectively time-independent.

The remaining of the paper is organized, as follows. The system under study is defined in Section 2 from the perspective of the FPE. Section 3 presents the concept of a non-normalizable quasi-equilibrium, and its characteristic phenomenology. Section 4 shows the derivation of the approximate solution of the FPE in the intermediately long-time limit, based on the eigenfunction expansion of the solution. Section 5 describes the regularization procedure. Section 6 describes the implications for quasi-equilibrium.

## 2. The System

We consider the overdamped dynamics of a Brownian particle in one dimension, which is governed by the Langevin equation (LE) [6,7]

(1) $$\begin{array}{c} \gamma \frac{dx}{dt}=F\left(x\right)+\;\sqrt{2\gamma k_{B}T}\;\eta \left(t\right), \end{array}$$

where γ is the damping coefficient, η(t) is a Gaussian white noise with zero mean and variance $\langle \eta \left(t\right)\eta \left(t^{\prime }\right)\rangle =\;\delta \left(t-t^{\prime }\right)$, and $F\left(x\right)=-\partial_{x}V\left(x\right)$ is the force. We assume, crucially, that the potential V(x) has a well at the origin and it is flat for large *x*. Alternatively, we can investigate the associated FPE [6,7] for the probability density function (PDF) P(x,t), namely,

(2) $$\frac{\partial }{\partial t}P\left(x,t\right)=D\left\{\frac{\partial^{2}}{\partial x^{2}}-\frac{\partial }{\partial x}\frac{F(x)}{k_{B}T}\right\}P\left(x,t\right),$$

where $D=k_{B}T/\gamma$ is the diffusion coefficient.

Both of the perspectives yield, in principle, the same results, and the PDF that solves the FPE is obtained by averaging over trajectories $x_{t}\equiv x\left(t\right)$ that are solutions of the LE, i.e.,

(3) $$\begin{array}{c} P\left(x,t\right)={\langle \delta \left(x-x_{t}\right)\rangle }_{p}\;, \end{array}$$

where ${\langle \ldots \rangle }_{p}$ represents an average taken over all possible paths $x_{t}$ and δ is the Dirac delta function. Other observables, such as the MSD, can also be evaluated as $\langle x^{2}\left(t\right)\rangle ={\langle x_{t}^{2}\rangle }_{p}$.

Note that the Boltzmann-Gibbs (BG) solution

(4) $$P\left(x\right)=\frac{1}{Z}\;e^{-V\left(x\right)/\left(k_{B}T\right)}$$

would be a stationary solution of the FPE Equation (7) if the potential were confining. However, this expression will not work for the cases studied here, as it is not normalizable. This is because the diffusion cannot be blocked indefinitely in a potential that is flat at long distances (V(x)→0 for x±∞). As a paradigm for this kind of potential, let us consider the families

(5) $$V_{\mu }\left(x\right)=-\frac{U_{0}}{{\left(1+{\left(x/x_{0}\right)}^{2}\right)}^{\frac{\mu }{2}}},$$

(6) $$V_{\kappa ,\mu }\left(x\right)=-\frac{U_{0}}{2}\;\frac{1+\cos(\kappa \;x/x_{0})}{{\left(1+{\left(x/x_{0}\right)}^{2}\right)}^{\frac{\mu }{2}}},$$

with μ>0 and κ a real parameter. Moreover, for simplicity, we have assumed even functions.

**Figure 1:**
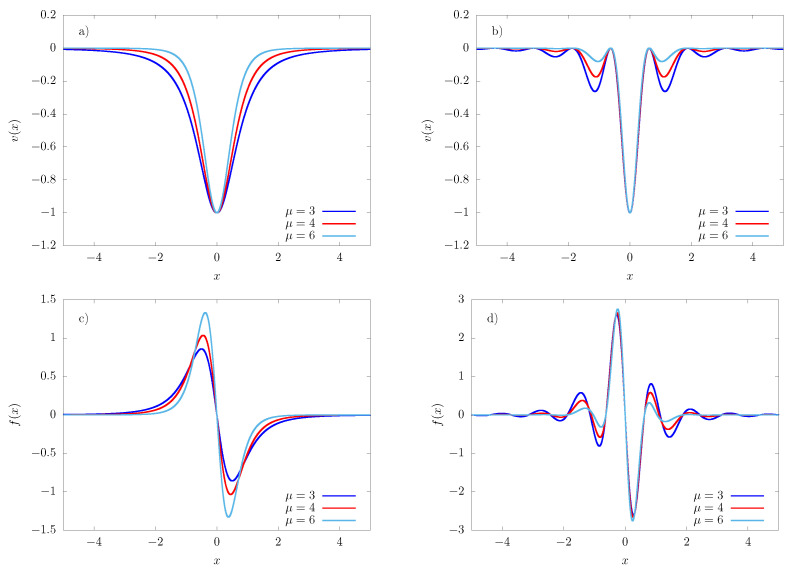
Dimensionless potentials (**a**) $v_{\mu }\left(x\right)$ and (**b**) $v_{\kappa ,\mu }\left(x\right)$, (**c**,**d**) the respective forces, plotted for three different values of μ and κ=5. Notice that the potential becomes flat and the force falls to zero at large distances from the origin and, therefore, ineffective, in the sense that these fields are non-binding and the normalization factor *Z* in Equation (4) diverges.

It is useful to use dimensionless variables. Subsequently, we adopt the lengthscale $x_{0}$, which represents the effective region of the potential well, the timescale $t_{0}=x_{0}^{2}/D$ related to free diffusion over this lengthscale, and the energy scale $U_{0}$ representing the well depth. Dimensionless versions of both potential and force can be defined as $v\left(x\right)=V\left(x\right)/U_{0}$ and $f\left(x\right)=x_{0}F\left(x\right)/U_{0}$, respectively. A scaled temperature can also be defined as the ratio between the thermal energy and the well depth $\xi =k_{B}T/U_{0}$. After the change of variables $x/x_{0}\to x$, $t/t_{0}\to t$, the scaled FPE equation becomes

(7) $$\begin{array}{c} \frac{\partial }{\partial t}P\left(x,t\right)=\frac{\partial^{2}}{\partial x^{2}}P\left(x,t\right)-\frac{1}{\xi }\frac{\partial }{\partial x}\left(f(x)P(x,t)\right), \end{array}$$

where the paradigmatic potentials become

(8) $$v_{\mu }\left(x\right)=-\frac{1}{{\left(1+x^{2}\right)}^{\frac{\mu }{2}}},$$

(9) $$v_{\kappa ,\mu }\left(x\right)=-\frac{1}{2}\;\frac{1+\cos(\kappa \;x)}{{\left(1+x^{2}\right)}^{\frac{\mu }{2}}},$$

and $f\left(x\right)=-\partial_{x}v\left(x\right)$. Notice that v(0)=−1. The form of these potentials is illustrated in Figure 1 for different values of μ.

**Figure 2:**
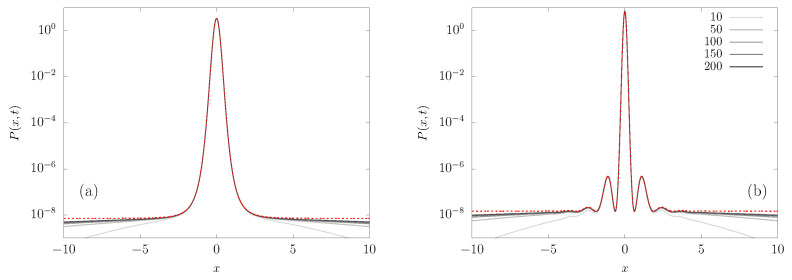
Probability density function P(x,t) for different times, from the numerical integration of the FPE (7) with the potentials (**a**) $v_{4}\left(x\right)$ and (**b**) $v_{5,4}\left(x\right)$, for ξ=0.05. For comparison, in each case we also plot (red dotted line) the Boltzmann expression that is given by Equation (47), $e^{-v(x)/\xi }/\left(2\ell_{0}\right)$, where $\ell_{0}$ is defined in Equation (25). The maxima in the plots correspond to minima in the potential field. Note that, as we increase time, the approximation given by Equation (47) works better; however, for times much longer than the escape time, a different behavior will be found.

The evolution of a packet of particles can be accessed by numerically integrating the FPE Equation (7) in order to obtain the PDF P(x,t). We employed a forward-time (explicit) centered in space scheme, based on a fourth-order Runge–Kutta method and second-order spatial discretization [8]. The initial condition has the particles starting at the origin, i.e., P(x,0)=δ(x). The PDF at different times, after the short initial transient, is shown in Figure 2 for the potentials $v_{4}\left(x\right)$ and $v_{5,4}\left(x\right)$. Figure 2 exhibits an interesting property, namely, the probability density is proportional to the Boltzmann factor exp(−v(x)/ξ), i.e., the extrema of the potentials in Figure 1 correspond to extrema of the density in Figure 2. Thus, here we already see that some concepts of BG statistics are still valid. Additionally, notice that most of the probability is in the central region, where the force is significantly non-null. That is, the area under the curve P(x,t) vs *x* in that central region is nearly one, while outside there are just rare fluctuations. This motivates the analysis of the FPE instead of finite sample Langevin simulations, as it would require an enormous amount of trajectories in order to accurately sample that region. However, from an experimentalist perspective, the central region is clearly the most important for computing the observables of interest.

## 3. Non-Normalizable Quasi-Equilibrium

To understand the dynamics of the Brownian particle, it is useful to investigate the time evolution of the mean square displacement (MSD), defined as

(10) $$\langle x^{2}\left(t\right)\rangle =\int_{-\infty }^{\infty }x^{2}P\left(x,t\right)\;dx,$$

which gives the fluctuation of the position, recalling that we start the particles on the origin and, from the symmetry of the problem (v(x)=v(−x)), 〈x〉=0 for all times.

**Figure 3:**
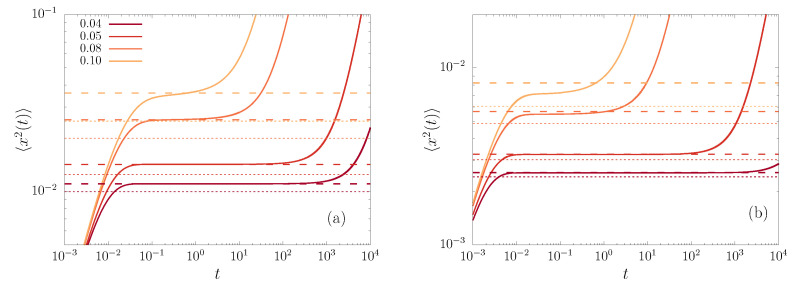
The MSD $\langle x^{2}\left(t\right)\rangle$ versus time *t*, obtained from numerical solutions of the FPE (solid lines) with the potential fields given by (**a**) $v_{4}\left(x\right)$ and (**b**) $v_{5,4}\left(x\right)$, and different values of ξ indicated in the legend. The dashed lines correspond to the expression derived along this work for NQE states, as given by Equation (57). Being drawn for comparison, the dotted lines correspond to the harmonic approximation ($\langle x^{2}\rangle =\xi /\mu$ and $\langle x^{2}\rangle =2\xi /\left(\kappa^{2}+2\mu \right)$), showing the improvement of the presented theory.

The time evolution of the MSD, as obtained by numerical integration of the FPE, is illustrated in Figure 3, for the potentials fields in Equations (8) and (9) with μ=4, at different values of ξ.

We observe a short-time increase of the MSD before the particles reach a quasi-equilibrium state (what we called NQE), where the MSD remains almost constant, yet, at even longer times, the particles escape the well and normal diffusion leads to an eventually linear increase of the MSD with time. The NQE state is long lived, and, intuitively, the deeper the well depth with respect to the temperature the longer is the lifetime of this stagnated state. This behavior is observed as well for thermodynamic observables, such as the energy or entropy, as previously shown [1].

**Figure 4:**
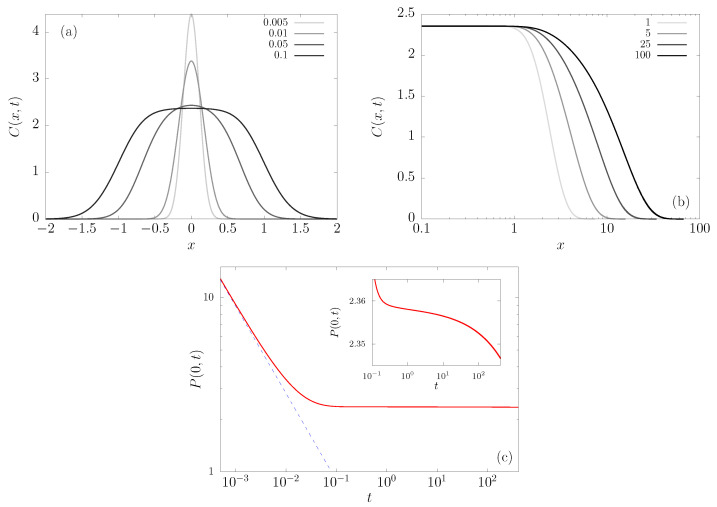
Pre-factor of the PDF, C(x,t) defined in Equation (11), at different times, for the field $v_{4}$ and scaled temperature ξ=0.1. (**a**) For very small times, (**b**) for an intermediate time scale. The behavior of the PDF at the origin P(0,t) is shown in (**c**), with its linear ordinate axis representation in the inset.

In order to better understand this phenomenon, it is useful to write the PDF factoring out the BG factor as

(11) $$P\left(x,t\right)=C\left(x,t\right)\;e^{-\{v(x)-v(0)\}/\xi }\;,$$

where the prefactor C(x,t) is plotted in Figure 4a as a function of *x* in the upper panels, for different times *t*. Notice that there is a large-*x* cut-off that diffuses away, while the central part flattens and attains an almost stationary level (see Figure 4b), namely C(x,t) becomes essentially constant in that region after a certain time. This means that the PDF approaches the shape that is defined by Equation (4), but the normalization is set by the region where the prefactor is flat.

In Figure 4c, we highlight the behavior of P(x,t) at the origin. For small times, we can see that $P\left(0,t\right)\propto 1/\sqrt{t}$ is a free diffusion until it reaches an approximately constant value, while the inset highlights how the value is still decreasing, albeit very slowly. This is valid for times that are long compared to the relaxation in the well but shorter than the Arrhenius escape time, of order $e^{1/\xi }$, which we will call below intermediate times, but the point is that in experimental situations they can be very long indeed.

Now, besides the MSD, which is given by Equation (10), we consider its time derivative, evaluated as

(12) $$\begin{array}{ccc} \frac{d}{dt}\langle x^{2}\left(t\right)\rangle & = & \int x^{2}\frac{\partial P(x,t)}{\partial t}\;dx. \end{array}$$

Using the FPE (7), we can expand the expression of the time derivative of the MSD, as

(13) $$\begin{array}{c} \frac{d}{dt}\langle x^{2}\left(t\right)\rangle =\int x^{2}\frac{\partial P(x,t)}{\partial t}\;dx=\int x^{2}\left\{\frac{\partial^{2}}{\partial x^{2}}P\left(x,t\right)-\frac{1}{\xi }\frac{\partial }{\partial x}\left(f(x)P(x,t)\right)\right\}\;dx, \end{array}$$

and perform integration by parts to obtain

(14) $$\begin{array}{c} \frac{d}{dt}\langle x^{2}\left(t\right)\rangle =2+\langle xf(x)\rangle =2-\langle x\frac{\partial v}{\partial x}\rangle . \end{array}$$

This result allows for a qualitative description of the dynamics of a packet of particles starting at x=0, i.e., setting P(x,0)=δ(x). (See Figure 3).

(i) For very short times, the particles spread diffusely, the second term in Equation (14) is null and the force is not yet felt, since, by assumption, the force vanishes at the origin.

(ii) Once the particles have diffused enough, the influence of the force becomes relevant and slows down the diffusive process. Subsequently, there is a time where the value of the derivative in the left-hand side of Equation (14) becomes minimal and, if the temperature is small enough (ξ≪1), then the system attains a stationary-like regime, where $d\langle x^{2}\left(t\right)\rangle /dt\approx 0$, as shown in Figure 3 (see also Reference [1]). This means that $\langle x\partial_{x}v\rangle \approx 2$, which implies that the virial theorem becomes approximately valid.

(iii) Because the force is finite and vanishes for large *x*, it is unable to block the diffusion indefinitely, so after a time that can be exponentially large (that is, proportional to the Arrhenius factor $e^{U_{0}/k_{B}T}=e^{1/\xi }$), a significant fraction of particles escape to diffuse outside the well. In such case, the second term in Equation (14) becomes null again, which gives rise to the linear growth of the MSD.

## 4. Time-Dependent Solution

In this section, we go through the derivation of the time-dependent PDF, P(x,t) for intermediate times, over which the prefactor C(x,t) defined in Equation (11) is effectively constant in the central region of the system, as can be seen in Figure 2a. That is, as commented above, for times that are long when compared to the relaxation in the well, but shorter than the Arrhenius escape time [9,10,11].

The derivation is structured just as in the non-deep potential case [12,13]. Similar analyses of the time-dependent FPE were performed in the context of a logarithmic potential [5], and front propagation [14,15]. However, the existence of an intermediate temporal regime is new and its origin needs to be explained. We start from the FPE for the PDF P(x,t), as given by Equation (7). The observation of a quasi-stationary regime, as depicted in Figure 3, leads one to assume a time-independent solution in an intermediate long-timescale. Subsequently, we set the left-hand side of the FPE to zero, and we obtain the time-independent solution, which we call I(x),

(15) $$\begin{array}{c} I\left(x\right)=e^{[v(0)-v(x)]/\xi }. \end{array}$$

This solution, which is Boltzmaniann, satisfies the no-flux boundary condition ${\left[v^{\prime }\left(x\right)I\left(x\right)\right]}^{\prime }=0$. However, this solution is not normalizable. We use a mathematical trick in order to circumvent this difficulty. We put the system in a box of size 2L, where *L* (measured in units of $x_{0}$) is much larger than the effective region of the potential well, i.e., L≫1. The introduction of these walls at x=±L will allow for us to normalize the solution. On the other hand, heuristically, the particles will diffuse more slowly than a free particle, so we may use the latter case as an upper bound in order to conclude that as long as $L\gg \sqrt{t}$, the walls are totally irrelevant. On this timescale, our boxed model will be identical to the reality, where the particles are not limited in space.

The PDF P(x,t), for the initial condition P(x,0)=δ(x), can be written as the eigenfunction expansion [7],

(16) $$\begin{array}{c} P\left(x,t\right)=e^{v(0)/\xi }\left\{I\left(x\right)N_{0}^{-1}+\sum_{\left\{k\right\}}N_{k}^{-1}\Psi_{k}\left(0\right)\Psi_{k}\left(x\right)e^{-k^{2}t}\right\}, \end{array}$$

where *k* is a wavenumber (scaled by $1/x_{0}$) that is given by the no-flux boundary condition at x=±L, and $N_{k}$ is the normalization constant that is associated to the eigenfucntion $\Psi_{k}\left(x\right)$, with the zero-mode $\Psi_{0}\left(x\right)=I\left(x\right)$. Notice that, in the limit of large *L*, the eigenvalues spectrum becomes continuous, since the potential is non-binding; in essence, this is the same as the spectrum of a free particle. This free particle spectrum has also been observed for other potentials [4,5].

We set the normalization of $\Psi_{k}\left(x\right)$ via the condition $\Psi_{k}\left(0\right)=1$, so that

(17) $$\begin{array}{c} N_{0}=2\int_{0}^{L}I^{2}\left(x\right)\;e^{v(x)/\xi }dx=2\;e^{2v(0)/\xi }\int_{0}^{L}e^{-v(x)/\xi }dx, \end{array}$$

and

(18) $$\begin{array}{c} N_{k}=2\int_{0}^{L}\Psi_{k}^{2}\left(x\right)e^{v(x)/\xi }dx. \end{array}$$

By using the FPE, we find that the eigenfunctions $\Psi_{k}\left(x\right)$ satisfy [7]

(19) $$\begin{array}{c} \Psi_{k}^{\prime \prime }\left(x\right)+\frac{1}{\xi }{\left(v^{\prime }\left(x\right)\Psi_{k}\left(x\right)\right)}^{\prime }=-k^{2}\Psi_{k}\left(x\right). \end{array}$$

The leading zero-mode term of the expansion in Equation (16) is simply the Boltzmann steady state in a box (−L,L). The intermediate-long-time limit is clearly dominated by the small-*k* modes, since the larger ones are suppressed as far as $e^{-k^{2}t}\ll 1$. Accordingly, it is enough to only consider the small-*k* modes.

We need to treat two regimes separately; first, the range x≪1/k, where the right-hand side of Equation (19) is always small, denoted region **I**; and second, for x≫1 (region **III**). These two asymptotic limits must be matched in the overlap region 1≪x≪1/k (region **II**).

In region **I**, the term $-k^{2}\Psi_{k}\left(x\right)$ is negligible, due to the smallness of *k*. To leading order, we have the homogeneous equation,

(20) $$\begin{array}{c} \Psi_{k}^{\prime \prime }\left(x\right)+\frac{1}{\xi }{\left(v^{\prime }\left(x\right)\Psi_{k}\left(x\right)\right)}^{\prime }=0, \end{array}$$

with the zero-mode solution I(x). To next order, we write $\Psi_{k}\left(x\right)∼I\left(x\right)\left(1-k^{2}g\left(x\right)\right)$. Plugging this ansatz into Equation (19), we obtain

(21) $$\begin{array}{c} -v^{\prime }\left(x\right)g^{\prime }\left(x\right)+g^{\prime \prime }\left(x\right)=1. \end{array}$$

The boundary conditions translate to $g\left(0\right)=g^{\prime }\left(0\right)=0$, and so a simple calculation yields

(22) $$\begin{array}{c} g\left(x\right)=\int_{0}^{x}e^{v(x_{1})/\xi }\left(\int_{0}^{x_{1}}e^{-v(x_{2})/\xi }dx_{2}\right)dx_{1}\;. \end{array}$$

We will soon analyze the large *x* behavior of g(x) and for that purpose we define

(23) $$g_{1}\left(x\right)\equiv \int_{0}^{x}e^{-v(x^{\prime })/\xi }dx^{\prime }\;.$$

Assuming that v(x) falls fast enough at large *x* (faster than 1/x, i.e., μ>1 for the families of potentials we consider), then, for large *x*,

(24) $$\begin{array}{ccc} g_{1}\left(x\right) & = & \int_{0}^{x}\left(e^{-v(x)/\xi }-1+1\right)dx\\ & = & x+\int_{0}^{\infty }\left(e^{-v(x)/\xi }-1\right)dx-\int_{x}^{\infty }\left(e^{-v(x)/\xi }-1\right)dx\\ & \approx & x+\ell_{0}\;, \end{array}$$

where $\ell_{0}$ is related to the second virial coefficient from the theory of gases [16], and the integrand is essentially the Mayer f-function, namely,

(25) $$\begin{array}{c} \ell_{0}\equiv \int_{0}^{\infty }\left(e^{-v(x)/\xi }-1\right)dx\;, \end{array}$$

which is exponentially large, of order $e^{1/\xi }$, recalling that v(0)=−1. For instance, for a square well, straightforwardly, $\ell_{0}\propto \left(e^{1/\xi }-1\right)$, for a smooth potential, according to the harmonic approximation $\ell_{0}\approx e^{1/\xi }\sqrt{\frac{\pi \xi }{2v^{\prime \prime }\left(0\right)}}$. Additionally, in Equation (17), $N_{0}\approx 2\ell_{0}$, which, as we will see, will play the role of a regularized partition function. Recall that *v* falls faster than 1/x, so that the integral converges. Now, for large *x*,

(26) $$\begin{array}{ccc} g(x) & = & \int_{0}^{x}\left(e^{v(x_{1})/\xi }g_{1}\left(x_{1}\right)-x_{1}-\ell_{0}+x_{1}+\ell_{0}\right)dx_{1}\\ & = & \frac{x^{2}}{2}+\ell_{0}x+\int_{0}^{\infty }\left(e^{v(x_{1})/\xi }g_{1}\left(x_{1}\right)-x_{1}-\ell_{0}\right)dx_{1}\\ & & -\int_{x}^{\infty }\left(e^{v(x_{1})/\xi }g_{1}\left(x_{1}\right)-x_{1}-\ell_{0}\right)dx_{1}\\ & \approx & \frac{x^{2}}{2}+\ell_{0}x+A\;, \end{array}$$

where

(27) $$\begin{array}{c} A\equiv \int_{0}^{\infty }\left(e^{v(x)/\xi }g_{1}\left(x\right)-x-\ell_{0}\right)dx\;. \end{array}$$

This behavior of g(x) can be seen to be consistent with Equation (21).

**Figure 5:**
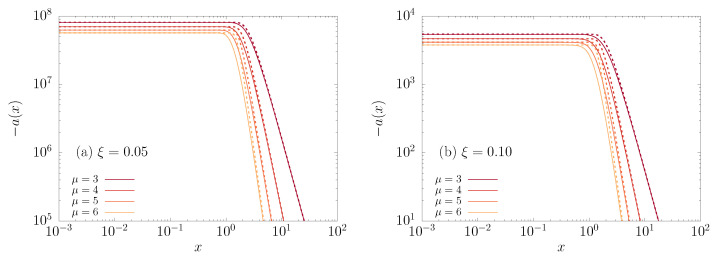
The integrand a(x) of A, for different values of ξ using the field $v_{\mu }\left(x\right)=-1/{\left(1+x^{2}\right)}^{\mu /2}$, with several values of μ. Solid lines represent a direct numerical evaluation of a(x) and the dotted line represents our approximation in Equation (29).

The next question is how the deepness of the potential affects $\ell_{0}$ and A. The calculation of A is a bit more challenging. With regard to the integrand of its definition, $a\left(x\right)=e^{v(x)/\xi }g\left(x\right)-x-\ell_{0}$, its value at 0 is $-\ell_{0}$, which, as we have already seen, is exponentially large. It is basically constant until some $x_{*}$, and then it decays as a power-law, as shown in Figure 5.

For large *x*, assuming that $v\left(x\right)\approx -1/x^{\mu }$, with μ>1, we have

(28) $$\begin{array}{c} a\left(x\right)\approx e^{-\frac{1}{\xi \;x^{\mu }}}\left(x+\ell_{0}+\frac{1}{\xi \left(\mu -1\right)x^{\mu -1}}\right)-x-\ell_{0}\;. \end{array}$$

The largest terms by far are those proportional to $\ell_{0}$, and so

(29) $$\begin{array}{c} a\left(x\right)\approx \ell_{0}\left(e^{-\frac{1}{\xi \;x^{\mu }}}-1\right). \end{array}$$

**Figure 6:**
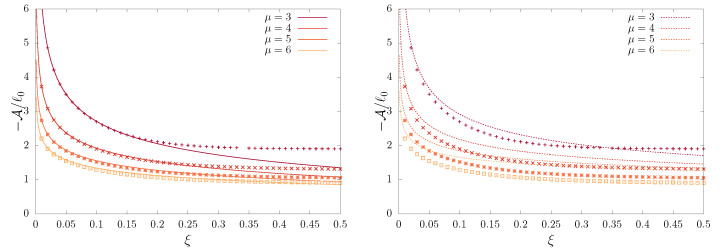
The ratio $\phi =-A/\ell_{0}$ vs. ξ, the scaled temperature of the system, where A and $\ell_{0}$ are given by Equations (27) and (25), respectively. We plot the direct solution (points) with the leading order (**right panel**, dotted lines), Equation (30), and the leading order with the first correction (**left panel**, solid line), Equation (32).

We show this approximation for $v\left(x\right)=-1/{\left(1+x^{2}\right)}^{\mu /2}$ in Figure 5. Integrating a(x), we find

(30) $$\begin{array}{c} A\approx -\frac{\ell_{0}}{\xi^{1/\mu }}\Gamma \left(\frac{\mu -1}{\mu }\right)\; \end{array}$$

so that A is exponentially large and it has the opposite sign of $\ell_{0}$ (see Figure 6). For our example, we can calculate the next correction as well, and

(31) $$\begin{array}{c} a\left(x\right)\approx \ell_{0}\left\{e^{-\frac{1}{\xi \;x^{\mu }}}\left(1+\frac{\mu }{2\xi x^{\mu +2}}\right)-1\right\} \end{array}$$

and

(32) $$\begin{array}{c} A\approx -\ell_{0}\left\{\frac{1}{\xi^{1/\mu }}\Gamma \left(\frac{\mu -1}{\mu }\right)-\frac{\xi^{1/\mu }}{2}\Gamma \left(\frac{\mu +1}{\mu }\right)\right\}\;. \end{array}$$

In the matching region **II**, where 1≪x≪1/k, based on Equation (19),

(33) $$\begin{array}{c} \Psi_{k}^{\mathrm{II}}\left(x\right)\approx e^{v(0)/\xi }\left(1-k^{2}\left(\frac{x^{2}}{2}+\ell_{0}x+A\right)\right). \end{array}$$

Note that, as long as *x* is not too large, the last two terms are dominant.

In region **III**, since x≫1, the $v^{\prime \prime }\left(x\right)$ and $v^{\prime 2}\left(x\right)$ terms are negligible and, therefore, Equation (19) now reads

(34) $$\begin{array}{c} \frac{\partial^{2}}{\partial x^{2}}\Psi_{k}\left(x\right)∼-k^{2}\Psi_{k}\left(x\right). \end{array}$$

When comparing to the region **II** solution, we get

(35) $$\begin{array}{c} \Psi_{k}^{\mathrm{III}}\left(x\right)\approx e^{v(0)/\xi }\left\{\cos\left(kx\right)-k\ell_{0}\sin\left(k\left(x-\phi \right)\right)\right\}\;, \end{array}$$

where

(36) $$\phi =-A/\ell_{0}\approx \Gamma \left(3/4\right)/\xi^{1/4}\;.$$

Let us remark that this derivation is based on a Dirac delta function at the origin as initial condition; however, the shift ϕ is expected to be the same for other initial distributions where almost all particles are in the effective region of the potential.

In Equation (35), since the $k^{\prime }$s are of order 1/L, the sin term is dominant as long as $L\ll e^{v(0)/\xi }$. We can now calculate the normalization,

(37) $$\begin{array}{c} N_{k}\approx Lk^{2}\ell_{0}^{2}e^{2v(0)/\xi }. \end{array}$$

Thus, in region **III**, we have

(38) $$\begin{array}{c} P^{\mathrm{III}}\left(x,t\right)=e^{v(0)/\xi }\left\{I\left(x\right)N_{0}^{-1}+\sum_{\left\{k\right\}}N_{k}^{-1}\Psi_{k}^{\mathrm{III}}\left(x\right)\;e^{-k^{2}t}\right\}\;. \end{array}$$

For large *L*, the spectrum becomes continuous, then the sum over {k} transforms into an integral, $\sum_{k}\to \frac{L}{\pi }\int dk$. However, notice that, at the same time that $\sqrt{t}\ll L$, it must be $L\ll e^{1/\xi }$. This latter constraint is crucial, as it ensures that the Boltzmaniann central part of the PDF (equilibrium state) has almost unit weight relative to the tails (continuum states). We achieve this by preventing *L* from being too large (see bounded domain approach in [1]).

Replacing the sum in Equation (38) and doing the integral yields

(39) $$\begin{array}{c} P^{\mathrm{III}}\left(x,t\right)\approx \frac{1}{2\ell_{0}}\left\{1-\mathrm{erf}\left(\frac{x-\phi }{2\sqrt{t}}\right)\right\}=\frac{1}{2\ell_{0}}\;\mathrm{erfc}\left(\frac{x-\phi }{2\sqrt{t}}\right)\;. \end{array}$$

The shift ϕ, as given by Equation (36), in this free diffusion solution, is induced by the well. It delimits the region of the well that has to be overcome to escape.

Our calculation for intermediate-times rests fundamentally on the assumption that very little flux has yet escaped the well. We can calculate the time where this assumption breaks down by examining how much probability has flowed from region **I** to region **III**. By considering a point x=ℓ∼ϕ, as given by Equation (36), where regions **I** and **III** overlap, the whole probability in region **III** can be written as

(40) $$\begin{array}{ccc} \int_{\ell }^{\infty }P^{\mathrm{III}}\left(x,t\right)dx & = & \frac{1}{2\ell_{0}}\left\{\frac{2t^{1/2}}{\sqrt{\pi }}\;e^{-\frac{{\left(\ell -\phi \right)}^{2}}{4t}}+\left(\phi -\ell \right)\;\mathrm{erfc}\left(\frac{\ell -\phi }{2\sqrt{t}}\right)\right\}\\ & \approx & \frac{t^{1/2}}{\sqrt{\pi }\;\ell_{0}}+\frac{\phi -\ell }{2\ell_{0}}+O\left(t^{-1/2}\right)\;. \end{array}$$

The last line in Equation (40) is the large-*t* expansion. $\int_{\ell }^{\infty }P^{\mathrm{III}}\left(x,t\right)dx$ is small. Subsequently, we conclude that the intermediate-long-time limit holds for times *t*, such that

(41) $$\begin{array}{c} \sqrt{t}\ll \sqrt{\pi }\ell_{0}\propto e^{-v(0)/\xi }=e^{1/\xi }\;, \end{array}$$

which is the Arrhenius factor [9,10,11]. Let us remark that, unlike Kramers’s escape problem, which is related to ours, here the potential field is flat at large *x*, and this makes the two problems non-identical.

To obtain an approximation for P(x,t) in region **I**, we need the small ξ approximation to the function g(x), see Equation (26). This works similarly to our small ξ approximation for A. We have

(42) $$\begin{array}{c} g\left(x\right)=\frac{x^{2}}{2}+\ell_{0}x+\int_{0}^{x}\left\{e^{v(x_{1})/\xi }\left(g_{1}\left(x_{1}\right)-x-\ell_{0}\right)+e^{v(x_{1})/\xi }\left(x_{1}+\ell_{0}\right)-x_{1}-\ell_{0}\right\}dx_{1}\;. \end{array}$$

In the integral over $x_{1}$, the first term within the brackets is clearly not exponentially large, and so the only terms proportional to $\ell_{0}$ are

(43) $$\begin{array}{c} g^{I}\left(x\right)\approx \ell_{0}\left(x-\int_{0}^{x}\left(e^{v(y)/\xi }-1\right)dy\right)\;. \end{array}$$

**Figure 7:**
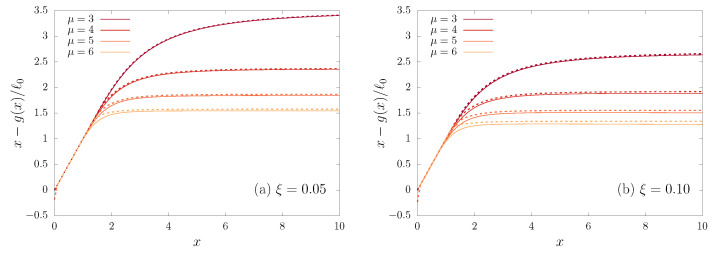
The ratio $x-g\left(x\right)/\ell_{0}$ vs. *x* calculated directly (solid line) from Equation (22), together with our deep well approximation (dotted line), Equation (45) for $v_{\mu }\left(x\right)=-1/{\left(1+x^{2}\right)}^{\mu /2}$ with several values of μ and two values of ξ.

For our first standard example, $v\left(x\right)=-1/{\left(1+x^{2}\right)}^{\mu /2}$, we have

(44) $$\begin{array}{ccc} g^{I}\left(x\right) & \approx & \ell_{0}\left\{x+\int_{0}^{x}\left(e^{-\frac{1}{\xi \;y^{\mu }}}\left(1+\frac{\mu }{2\xi y^{\mu +2}}\right)-1\right)dy\right\} \end{array}$$

(45) $$\begin{array}{ccc} \; & = & \ell_{0}\left\{x-\frac{x}{\mu }E_{1+1/\mu }\left(\frac{1}{\xi x^{\mu }}\right)+\frac{\xi^{1/\mu }}{2}\Gamma \left(1+1/\mu ,\frac{1}{\xi x^{\mu }}\right)\right\}\;, \end{array}$$

where $E_{n}\left(x\right)$ is the exponential integral function and Γ(n,x) is the incomplete Gamma function, outcomes of the calculation of the integral in Equation (44) [17]. This is verified in Figure 7, where we plot $\left(x-g(x)\right)/\ell_{0}$ vs. *x*, together with our analytic approximation.

Thus,

(46) $$\begin{array}{ccc} P^{I}\left(x,t\right) & \approx & \frac{I(x)}{2e^{v(0)}\ell_{0}}\left(1-g^{I}\left(x\right)\int_{0}^{\infty }\frac{dk}{\pi \ell_{0}}e^{-k^{2}t}\right)\\ & \approx & \frac{e^{-v(x)/\xi }}{2\ell_{0}}\left(1-\frac{1}{\sqrt{\pi t}}\frac{g^{I}\left(x\right)}{\ell_{0}}\right), \end{array}$$

which overlaps, as it must, with the region **III** result. For sufficiently large *t*, Equation (46) becomes

(47) $$\begin{array}{c} P^{I}\left(x,t\right)\approx e^{-v(x)/\xi }/\left(2\ell_{0}\right)\;, \end{array}$$

which is time-independent. Notice that, as mentioned earlier, $2\ell_{0}$ serves as an effective partition function, which replaces *Z* in Equation (4). Moreover, region **I** is where most of the probability is found and, hence, this expression captures the behavior of the majority of the particles.

**Figure 8:**
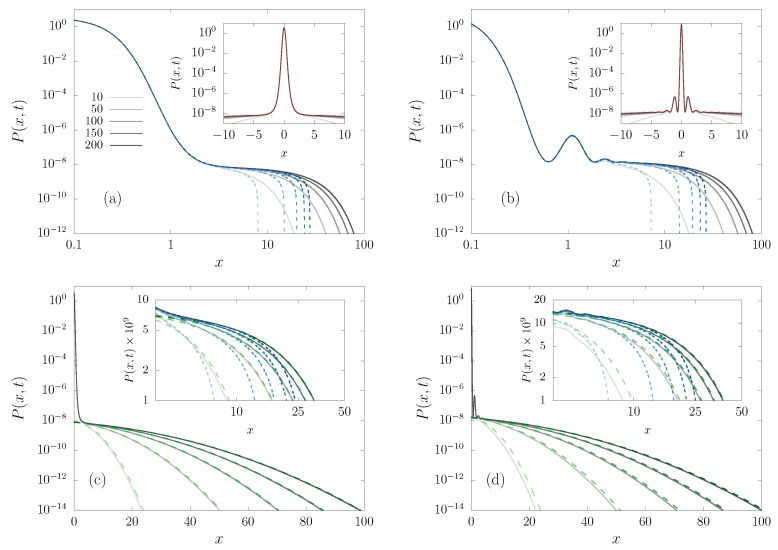
Central region **I** (**a**,**b**) and tail region **III** (**c**,**d**) of the PDF, for the potentials $v_{4}$ (**a**,**c**) and $v_{4,5}$ (**b**,**d**), at different times *t* (increasing from light to dark color) indicated in the legend. PDF from the numerical integration of the FPE (black solid lines) and theoretical predictions (dashed lines): $P^{I}\left(x,t\right)$ (blue short dashed) given by Equation (46), and $P^{III}\left(x,t\right)$ (green long dashed) that is given by (39). The upper insets include the Boltzmannian (red dashed) curve for comparison (duplicating Figure 2). The intermediate matching region is amplified in the lower insets.

Figure 8 summarizes the behavior of the PDF in regions **I** (center) and **III** (tail). (The upper insets replicate Figure 2, to complete the portrait.) The results from numerical simulations are represented by solid lines, while the theoretical results for regions **I** (short dashed line) and **III** (long dashed lines) are also plotted, in good agreement in the respective regions. In the insets, all of the curves are plotted together, in a zoom of the matching region.

## 5. Regularization Procedure

Because Equation (47) is time-independent, the averages computed when the system is inside the intermediate time-scale epoch outlined in Section 4 become almost constant in time. Even though it is not possible to apply the regular BG equilibrium statistics, for systems where ξ is small, we can, through a regularization procedure, obtain time-independent averages, which we refer to as NQE averages. We now demonstrate the regularization procedure that allows us to compute these averages.

The average of a given observable O(x) can be obtained by using the PDF as

(48) $$\begin{array}{ccc} \langle O\rangle & = & \int_{-\infty }^{\infty }O\left(x\right)\;P\left(x,t\right)\;dx\;. \end{array}$$

We can split the integration in two regions (x,ℓ) and (ℓ,∞), where *ℓ* is an intermediate length scale ($\ell ∼\phi =-A/\ell_{0}$, as defined above), namely,

(49) $$\begin{array}{ccc} \langle O\rangle & ≃ & 2\int_{0}^{\ell }O\left(x\right)\;P^{I}\left(x,t\right)\;dx+2\int_{\ell }^{\infty }O\left(x\right)\;P^{\mathrm{III}}\left(x,t\right)\;dx={\langle O\rangle }_{I}+{\langle O\rangle }_{\mathrm{III}}\;. \end{array}$$

Recalling that, for intermediate timescales, region **I** concentrates most of the probability and $P^{I}$ becomes nearly time-independent, then Equation (49) allows for the predicting of the NQE value. It is noteworthy that the NQE regime of different observables is related to different timescales.

We will now illustrate this procedure through the computation of the average MSD, as defined in Equation (10), for a system subject to the potential fields with a power-law decay. This is a simple, still good, example since the MSD constitutes a relevant dynamical measure of the spread of the particles around the central potential well, as defined in Equation (10).

First, we calculate ${\langle x^{2}\rangle }_{I}$, using (46). After neglecting the correction containing $g^{I}\left(x\right)$ for $t\gg \ell_{0}$, we perform the same trick that was used in Equation (24) to obtain $\ell_{0}$, namely,

(50) $$\begin{array}{ccc} & {\langle x^{2}\rangle }_{I} & ≃\frac{1}{\ell_{0}}\int_{0}^{\ell }x^{2}\;e^{-v(x)/\xi }\left(1-\frac{1}{\sqrt{\pi t}}\frac{g^{I}\left(x\right)}{\ell_{0}}\right)\;dx \end{array}$$

(51) $$\begin{array}{ccc} \; & ≃ & \frac{1}{\ell_{0}}\int_{0}^{\infty }x^{2}\;\left(e^{-v(x)/\xi }-h\left(x\right)\right)dx-\frac{1}{\ell_{0}}\int_{\ell }^{\infty }x^{2}\;\left(e^{-v(x)/\xi }-h\left(x\right)\right)dx+\\ & & +\frac{1}{\ell_{0}}\int_{0}^{\ell }x^{2}h\left(x\right)dx \end{array}$$

(52) $$\begin{array}{cc} ≃ & \frac{1}{\ell_{0}}\int_{0}^{\infty }x^{2}\;\left(e^{-v(x)/\xi }-h\left(x\right)\right)dx\;. \end{array}$$

Differently from the case of Equation (24), for integral convergence, we must set

(53) $$h\left(x\right)=\sum_{k=0}^{K}{\left(-1\right)}^{k}{\left(v\left(x\right)/\xi \right)}^{k}/k!\;,$$

where we sum up to the integer *K* defined, for the specific observable, as the minimal value to ensure that the integral converges. In the specific case of $x^{2}$, in Equation (50), we have K=⌊3/μ⌋, and, for a general $x^{n}$, we have K=⌊(n+1)/μ⌋. The reasoning behind this technique goes beyond merely creating a converging integral, for small values of ξ, we have $h\left(x\right)\ll e^{-v(x)/\xi }$ for the range (x,ℓ), therefore we are able to cure the diverging contribution from the tail whilst maintaining a very accurate result overall.

In particular, for μ>3, we have h(x)=1. The first term in Equation (51) is the only time-independent term, which is related to the standard BG probability. Recall that, from Equation (25), $\ell_{0}\equiv \int_{0}^{\infty }\left(e^{-v(x)/\xi }-1\right)dx$, of order $e^{1/\xi }$. The second term scales as 1/ξ, so that it becomes increasingly negligible when compared to $\ell_{0}$. The same occurs for the last term in Equation (51), which, for h(x)=1, becomes $\ell^{3}/\left(3\ell_{0}\right)$.

Now, we calculate ${\langle x^{2}\rangle }_{\mathrm{III}}$, by using Equation (39). In this case, it is possible to perform the integral exactly to obtain

(54) $$\begin{array}{ccc} {\langle x^{2}\rangle }_{\mathrm{III}} & = & \frac{1}{\ell_{0}}\int_{\ell }^{\infty }x^{2}\;\mathrm{erfc}\left(\frac{x-\phi }{2\sqrt{t}}\right)\;dx\\ & = & \frac{1}{3\sqrt{\pi }\;\ell_{0}}\left(2\sqrt{t}\;\left(\phi^{2}+\ell \phi +\ell^{2}+4t\right)\;e^{-\frac{{\left(\ell -\phi \right)}^{2}}{4t}}+\sqrt{\pi }\;\left(\phi^{3}-\ell^{3}+6\phi t\right)\;\mathrm{erfc}\left[\frac{\ell -\phi }{2\sqrt{t}}\right]\right)\\ & \approx & \frac{8t^{3/2}}{3\ell_{0}\sqrt{\pi }}+2\phi t+\frac{2\phi^{2}t^{1/2}}{\ell_{0}\sqrt{\pi }}+\frac{\phi^{3}-\ell^{3}}{3\ell_{0}}+O\left(t^{-1/2}\right)\;, \end{array}$$

where the last member of the equation is obtained from the large-*t* expansion.

Putting this all together, we write, up to the first correction for large time,

(55) $$\begin{array}{c} \langle x^{2}\left(t\right)\rangle ≃{\langle x^{2}\rangle }_{I}+{\langle x^{2}\rangle }_{\mathrm{III}}≃{\langle x^{2}\rangle }_{I}+8t^{3/2}/\left[3\sqrt{\pi }l_{0}\right]\;. \end{array}$$

The average will become almost time-independent for time-scales *t*, such that

(56) $$\begin{array}{c} t\ll \frac{{\left(3\ell_{0}\right)}^{2/3}}{4\pi^{1/3}}\propto e^{-2v(0)/3\xi }\;, \end{array}$$

which is also related to the Arrhenius factor [9,10,11]. The time-dependent contribution will be negligible and, for large times, we can estimate the departure times. Subsequently, the NQE average is estimated (when μ>3) as

(57) $$\begin{array}{c} {\langle x^{2}\rangle }_{\mathrm{NQE}}≃{\langle x^{2}\rangle }_{I}≃\frac{1}{\ell_{0}}\int_{0}^{\ell }x^{2}e^{-v(x)/\xi }dx\approx \frac{\int_{0}^{\infty }x^{2}\left(e^{-v(x)/\xi }-1\right)dx}{\int_{0}^{\infty }\left(e^{-v(x)/\xi }-1\right)dx}\;. \end{array}$$

The performance of these approximations can be appreciated in Figure 3, for different values of ξ. The smaller ξ, the longer the lifetime of the NQE regime and the better the theoretical prediction for the NQE level works, as given by Equation (57). The figure also exhibits the improvement of the theory for the NQE with respect to the harmonic approximation of the potential well (dotted lines).

For a general observable, and potentials decaying faster that 1/x (μ>1), the NQE average is

(58) $$\begin{array}{c} {\langle O\left(x\right)\rangle }_{\mathrm{NQE}}=\frac{\int_{0}^{\infty }O\left(x\right)\left(e^{-v(x)/\xi }-h\left(x\right)\right)dx}{\int_{0}^{\infty }\left(e^{-v(x)/\xi }-1\right)dx}\;, \end{array}$$

where h(x) is defined as Equation (53), ensuring convergence. Therefore, it is determined by the observable and the potential field [1]. For potentials with 0<μ≤1, $\ell_{0}$ must be modified; hence, the denominator in Equation (58) becomes

(59) $$\begin{array}{c} \ell_{0}=\int_{0}^{\infty }\left(e^{-v(x)/\xi }-l\left(x\right)\right)dx \end{array}$$

with

(60) $$l\left(x\right)=\sum_{k=0}^{K^{\prime }}{\left(-1\right)}^{k}{\left(v\left(x\right)/\xi \right)}^{k}/k!\;,$$

where $K^{\prime }=\left\lfloor 1/\mu \right\rfloor$.

## 6. Final Remarks

We have presented the solution of the FPE (7) for asymptotically flat potentials with a deep well at the origin, by using an eigenfunction expansion. In such potential fields, long-lived NQE states emerge, as heuristically shown in our previous work [1]. The non-confinement of the potential makes the standard partition function divergent, which hampers its direct application. Nevertheless, a regularization procedure is still possible, allowing one to calculate quantities in the NQE states along the lines of the recipes of statistical mechanics (see Equation (58)).

The spectrum of eigenvalues is continuous like that of a free particle, still the Boltzmann measure is preserved for intermediate times, such that $\sqrt{t}\ll \sqrt{\pi }\ell_{0}\propto e^{1/\xi }$, which is the Arrhenius time. In such case, according to Equations (39) and (47), the approximate solution that we found can be summarized as

(61) $$\begin{array}{c} P\left(x,t\right)=\left\{\begin{array}{cc} \frac{1}{2\ell_{0}}\;e^{-v(x)/\xi }, & \mathrm{region}I,\\ \frac{1}{2\ell_{0}}\;\mathrm{erfc}\left(\frac{x-\phi }{2\sqrt{t}}\right), & \mathrm{region}\mathrm{III}, \end{array}\right. \end{array}$$

where region **I** corresponds to the central part of the PDF and region **III** to the tails, while region **II** is where both solutions overlap, as can be seen in Figure 8; moreover, $2\ell_{0}$, as defined in Equation (25), is a lengthscale that plays the role of an effective partition function, and the shift ϕ can be estimated through Equation (36). Region **I** concentrates most of the probability, out of fluctuations in region **III**, where the erfc function acts as an effective cutoff blocking free diffusion. The shift is related to the region of the well that has to be overcome to escape. To see this note that $l_{0}$ is large, but $e^{-v(0)/\xi }=e^{1/\xi }$ is similarly large (while the erfc is of order one or less), hence the small *x* solution in region **I** exponentially overwhelms the solution in region **III**. Subsequently, the shift ϕ decreases with increasing scaled temperature ξ, as can be seen in Figure 6. Equation (61) shows how nearly time-independent solutions can emerge. They last exponentially long times, for sufficiently low temperatures, and they can be associated to the NQE regime.

The physics of non-normalizable states has been the object of extensive studies within infinite ergodic theory [18,19], in situations that are different from what we consider here. For example, in cases where the particle escapes and returns to the well many times, the density in region **I** decays in time, while, in our case, it remains nearly constant. Additionally, notice that the current approach differs from the calculation of ensemble averaged observables performed in the limit of t→∞ [12,13,20]. Here, we avoid the limit of infinite time by considering the upper bound on the measurement time, namely, the escape time, $e^{1/\xi }$, which allows us to isolate the dominant Boltzmann-like behavior at the center of the packet.

Furthermore, let us mention that preliminary results indicate that some form of ergodicity holds in NQE states. If we restrict the time integration over trajectories to the interval where the observable is in its plateau, i.e., after the short initial transient and for times shorter than the Arrhenius time, we will obtain the same result as the NQE ensemble average presented in Equation (58). NQE states can emerge in a wide range of systems and observables [1], beyond the MSD used here in our examples, as long as there is a clear separation of lengthscales between the effective well and long tail behavior. This indicates that the method is rather robust, in the sense that it is not restricted to potentials with a single well. For sufficiently separated wells, more than one plateau can emerge, in that case, the theory predicts the last one, before some particles escape the full region where forces are effective. It would be interesting to verify, in experimental settings, the existence of NQE, for example, while using single molecule tracking. Systems where there is a region with an effective potential well and non-confinement otherwise are, for instance, atoms in optical tweezers [21], and single molecules in solid-liquid interfaces [22,23,24].

The theory will also work in higher dimensions, although the details should be part of a separate study. The investigation of the fractional Fokker–Planck equation [25] for anomalous dynamics, as well as generalized Langevin equations with memory, and the underdamped dynamics would be interesting extensions of the present work.
